# T- and B-cell lymphomas in 2 captive green tree pythons

**DOI:** 10.1177/10406387251337369

**Published:** 2025-05-13

**Authors:** Sara Pagliarani, Luke Haydock, Leonardo Susta, Pauline Delnatte, Cédric B. Larouche, Mauricio Seguel

**Affiliations:** Department of Pathobiology, Ontario Veterinary College, University of Guelph, Guelph, Ontario, Canada; Department of Pathobiology, Ontario Veterinary College, University of Guelph, Guelph, Ontario, Canada; Department of Pathobiology, Ontario Veterinary College, University of Guelph, Guelph, Ontario, Canada; Toronto Zoo, Scarborough, Ontario, Canada; Toronto Zoo, Scarborough, Ontario, Canada; Department of Pathobiology, Ontario Veterinary College, University of Guelph, Guelph, Ontario, Canada

**Keywords:** B-cell lymphoma, CD3, green tree pythons, immunohistochemistry, *Morelia viridis*, neoplasm, PAX5, snakes, T-cell lymphoma

## Abstract

Two captive 19-y-old green tree pythons (*Morelia viridis*), a male and a female, were diagnosed with lymphoma. At autopsy, the female was in poor body condition and had an extensive subcutaneous soft tissue mass along the ribs. The mass was composed of numerous neoplastic round cells that were also present in the liver and spleen and within blood vessels. Neoplastic cells had marked, diffuse membranous-to-cytoplasmic immunolabeling for CD3, consistent with disseminated leukemic T-cell lymphoma. The male had a history of chronic spinal deformities and was in poor body condition. All examined organs contained variable numbers of neoplastic round cells with moderate-to-marked nuclear-to-cytoplasmic immunolabeling for PAX5, consistent with B-cell lymphoma. Here we describe 2 distinct immunophenotypes of lymphomas, including a B-cell neoplasm that has not been reported previously in snakes, to our knowledge. Our 2 cases highlight the potential value of CD3 and PAX5 immunohistochemical markers in ophidians and expand the spectrum of neoplastic diseases documented in reptiles. Understanding the clinical significance of lymphoma in snakes, including its potential impact on prognosis and challenges in management, is critical to refining therapeutic approaches in captive reptile care.

Historically considered a rare incident in reptiles, neoplasia is now commonly diagnosed in captive reptile medicine practice,^3^ with an incidence comparable with that of mammals and birds. The reported incidence of neoplasia in reptiles is 12–26% based on records of animals submitted to diagnostic pathology facilities.^2,3,5,12,14,19^ Retrospective studies of reptile neoplasia showed that the prevalence of neoplasia is highest in snakes, followed by lizards, chelonians, and crocodilians,^3,4^ and that, among the suborder *Serpentes*, colubrids are the most commonly reported, followed by crotalids, viperids, and boids. Neoplasia affecting the hematopoietic and lymphoid systems are frequently reported and are over-represented in some studies.^2,14^ In a 9-y retrospective study of neoplasia in reptiles, of 325 tumors identified in snakes, 33 (11%) were classified as lymphoma, including 10 cases in boids (1.2% prevalence).

Clinical signs reported in reptiles diagnosed with neoplasia were nonspecific, and included lethargy, anorexia, dyspnea, presence of masses, celomic distension, and constipation^3^; anemia and leukocytosis have also been reported in snakes diagnosed with lymphoma.^11,16^ However, most reports do not specify the immunohistochemical profile of these neoplasms, resulting in limited knowledge about the most common types of lymphoma in snakes. Here we describe the presentation of 2 immunophenotypes of lymphoma in 2 captive green tree pythons (*Morelia viridis*).

Both snakes were housed at a zoologic institution prior to submission for autopsy. All tissues collected were placed in 10% neutral-buffered formalin to allow adequate fixation and processed for light microscopy at the University of Guelph–Animal Health Laboratory (AHL; Guelph, Ontario, Canada). Immunohistochemistry (IHC) was performed on formalin-fixed paraffin-embedded tissue using the following antibodies: anti-human CD3 (rabbit polyclonal CD3; T-lymphocyte marker, clone/code A045201-2, Dako), anti-human PAX5 (mouse monoclonal; B-cell lineage–specific activator protein, clone/code CM207B, Biocare Medical), and anti-IBA1 (rabbit polyclonal; ionized calcium–binding adaptor molecule 1 expressed in macrophages, clone/code CP290A, Biocare Medical). IHC and Luna stain (only for case 1) were performed at the AHL using standard procedures and manufacturers’ instructions; however, these tests are validated for mammalian species and have not been specifically validated for reptiles.

Case 1, a 19-y-old female green tree python, had a history of constipation, cloacal prolapse, and abnormal respiratory sounds. This snake was captive-born and acquired when 1-y-old. During physical examination, the animal was in poor body condition and lethargic. CBC revealed marked leukocytosis (65.7 × 10^9^ cells/L; RI: 1.2–18.7 × 10^9^ cells/L^10^) and mild lymphocytosis (16.4 × 10^9^ cells/L, RI: 0.07–11.8 × 10^9^ cells/L^10^; 25% relative count). The serum biochemistry panel was unremarkable. Due to rapid clinical deterioration, euthanasia was elected. Gross postmortem examination revealed a regionally extensive, 10 × 3-cm dark-red soft mass effacing the subcutaneous tissue along the ribs on the left lateral thorax, ~45 cm cranial to the cloaca. Histologically, dense sheets of neoplastic round cells dissected between the ribs and the myofibers of the iliocostalis and longissimus dorsi muscle groups (Fig. 1A, 1B). The neoplastic cells were large, ~15–18-µm diameter, had indistinct cell borders, a scant amount of eosinophilic cytoplasm, a high nuclear:cytoplasmic ratio, single 7–10-μm round-to-oval-to-reniform nuclei with stippled chromatin, and a single magenta nucleolus (Fig. 1B). Anisocytosis and anisokaryosis were minimal, and there were 4 mitotic figures in 10 standardized 400× fields (2.37 mm^2^); ~10% of cells were necrotic. Admixed with the neoplastic cells were occasional solitary to densely clustered granulocytes with a round nucleus and eosinophilic cytoplasmic granules that stained bright pink with Luna stain, consistent with eosinophils. Moderate-to-large numbers of similar round neoplastic cells variably effaced, infiltrated, or expanded the hepatic sinusoids and portal tracts, the cortex and medulla of adrenal glands, and the renal interstitium. Numerous renal, serosal, and oviductal blood vessels contained intraluminal aggregates of large neoplastic round cells like those described in other organs. Over 90% of the neoplastic cells had moderate-to-marked cytoplasmic-to-membranous immunolabeling for CD3 (Fig. 1C). Scattered aggregates of cells, comprising <10% of the neoplastic cells, had diffuse nuclear immunolabeling with PAX5. These cells were consistently associated with regions of increased intratumoral B-cell infiltration (Fig. 1D). Occasional isolated cells comprising <5% of the neoplasm had marked cytoplasmic immunolabeling for IBA1, consistent with macrophages. The animal also had heterophilic salpingitis and rare neoplastic round cells in the oviduct lumen.

**Figure 1.**
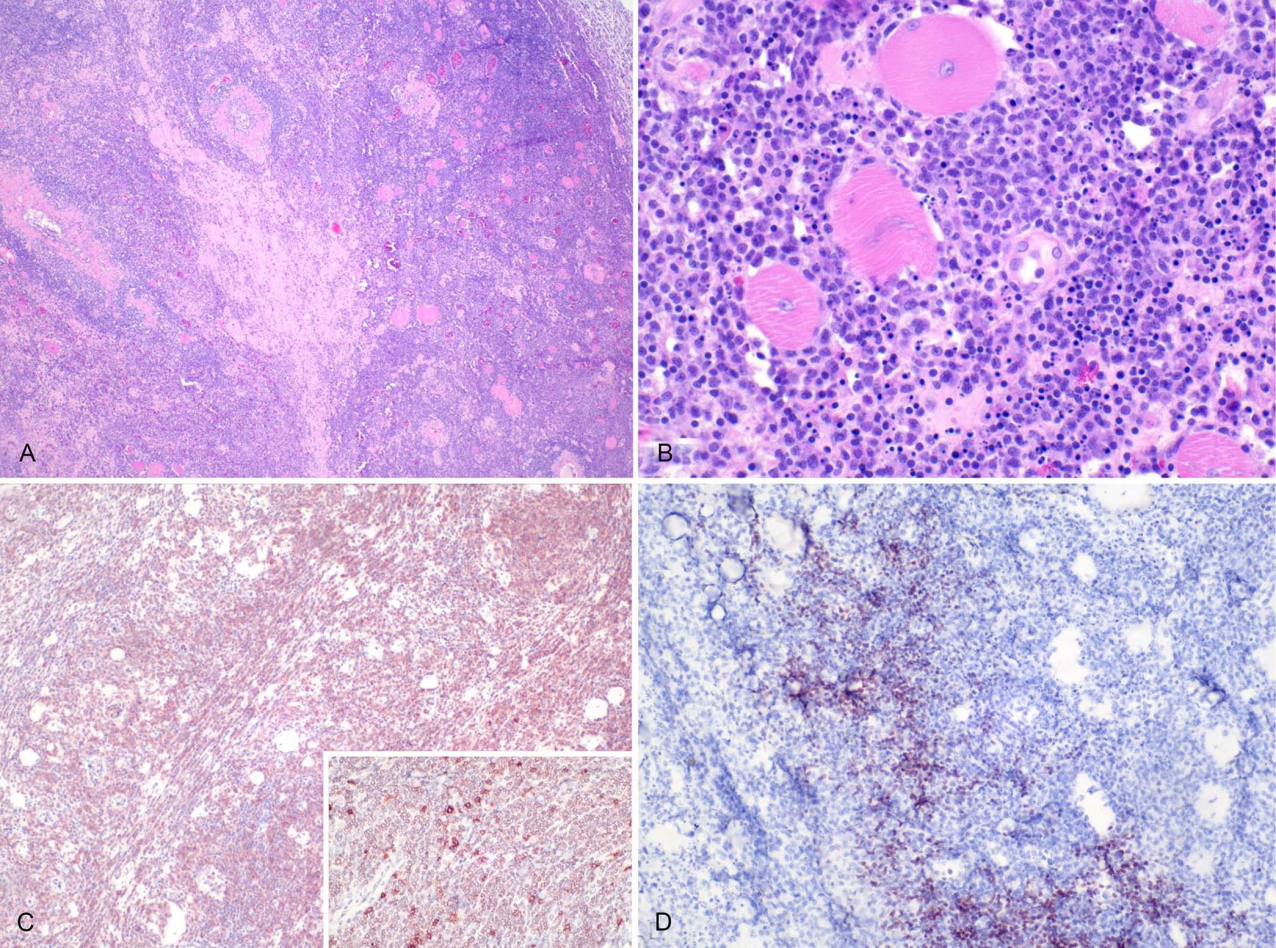
T-cell lymphoma in a 19-y-old female green tree python, case 1. **A, B.** Dense sheets of an infiltrative population of neoplastic round cells dissect between myofibers and soft tissue of the body wall. H&E. **B.** The neoplastic cells have poorly distinct cell borders, scant amounts of eosinophilic cytoplasm, and a high nuclear:cytoplasmic ratio. Anisocytosis and anisokaryosis are minimal. A few cells are necrotic, with accumulation of karyorrhectic and pyknotic debris. H&E. **C.** Neoplastic cells in the body wall have positive cytoplasmic-to-membranous immunolabeling to CD3 (inset, brown). Hematoxylin counterstain. **D.** Immunohistochemical nuclear labeling for PAX5 in scattered aggregates of cells, comprising <10% of neoplastic cells and consistently associated with regions of increased granulocytic infiltration. Hematoxylin counterstain.

Case 2, a 19-y-old captive-born male green tree python, had a chronic history of spinal deformities that were present before arrival at the zoologic institution 5 y earlier. The animal repeatedly fell due to loose coiling. CBC revealed marked leukocytosis (30 × 10^9^ cells/L; RI: 1.2–18.7 × 10^9^ cells/L^10^) with mild lymphocytosis (18 × 10^9^ cells/L, RI 0.07–11.8 × 10^9^ cells/L^10^; 60% relative count). Due to sustained health decline, euthanasia was elected. On postmortem examination, in the cranial spinal segment, bone remodeling was marked in the dorsal aspect of most vertebral bodies; some vertebral bodies had a dark-brown, friable, necrotic core. Histologically, in the esophagus, stomach, and small intestine, a poorly defined, infiltrative round-cell neoplasm expanded the submucosa and extended into portions of the mucosa (Fig. 2A). The neoplastic cells were round, ~10–15-µm diameter, had distinct cell borders, and a single round, 5–7-µm central nucleus with condensed chromatin and a rim of eosinophilic cytoplasm. Anisocytosis and anisokaryosis were minimal, and there were 6 mitotic figures in 10 standardized 400× fields (2.37 mm^2^). Moderate numbers of similar neoplastic cells variably effaced, infiltrated, or expanded the pulmonary and myocardial interstitium. Throughout the gastrointestinal tract, occasional dense colonies of gram-negative bacilli were embedded in eosinophilic homogeneous material surrounded by numerous foamy macrophages and fewer heterophils (heterophilic granulomas). Over 90% of the neoplastic cells had marked nuclear immunolabeling for PAX5 (Fig. 2B, 2C). Within the neoplasm, <10% of round cells had marked membranous immunolabeling for CD3 (Fig. 2D), similar to lymphocytes surrounding granulomas in the gastrointestinal tract and in the hepatic portal triads.

**Figure 2.**
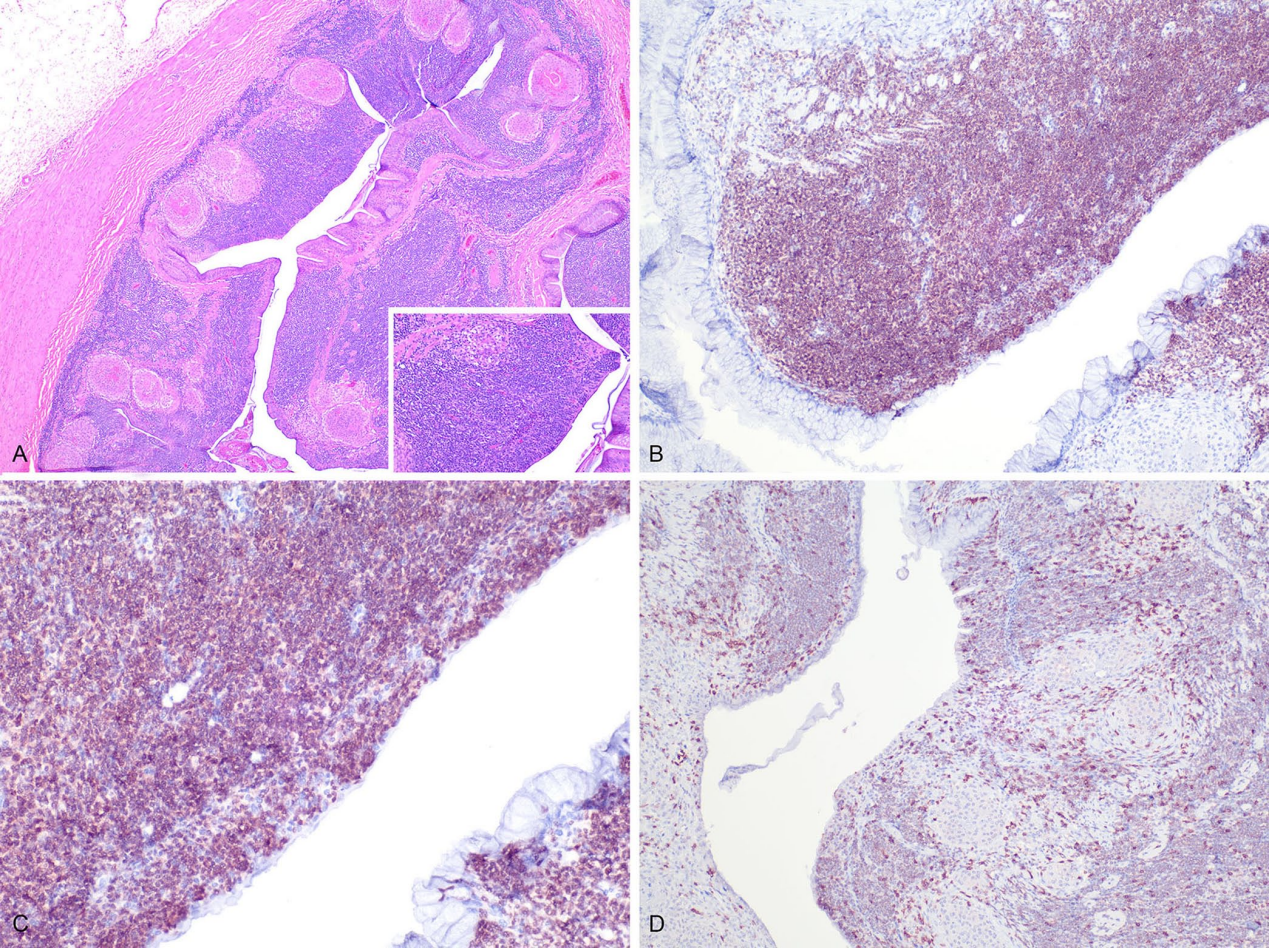
B-cell lymphoma in a 19-y-old male green tree python, case 2. **A.** Neoplastic round cells efface and expand the gastric mucosa and submucosa (inset). The submucosa is expanded by multifocal heterophilic granulomas. H&E. **B.** Neoplastic cells in the gastric epithelium have positive (brown) intranuclear immunolabeling to PAX5. Hematoxylin counterstain. **C.** High magnification of PAX5 immunolabeling (brown) demonstrating strong nuclear positivity in neoplastic lymphocytes. PAX5, a B-cell lineage–specific transcription factor, confirms the B-cell origin of the neoplastic population. Note the distinct nuclear staining pattern, consistent with the expected localization of PAX5 expression. Hematoxylin counterstain. **D.** Scattered non-neoplastic lymphocytes have positive cytoplasmic immunolabeling for CD3 (brown). Lymphocytes surrounding granulomas have strong cytoplasmic immunolabeling for CD3 (internal positive control). Hematoxylin counterstain.

In the bodies of the remodeled vertebrae was moderate chronic histiocytic and heterophilic osteomyelitis. It is important to clarify that bacterial cultures in this case did not yield *Salmonella* spp. Although *Salmonella* spp. have been associated with chronic vertebral lesions in reptiles, we did not explicitly test for *Salmonella* spp. via PCR or other targeted methods. Our bacterial culture identified *Ochrobactrum* spp., *Brevibacterium casei*, and *Corynebacterium* spp. from the necrotic vertebrae, suggesting a potential primary or secondary role for these organisms in lesion development. Although a pre-existing condition involving *Salmonella* spp. or other bacteria cannot be excluded, future studies could benefit from targeted *Salmonella* spp. cultures or molecular testing to clarify its potential involvement in similar cases.

We found immunophenotypically different lymphoid neoplastic processes consistent with T- and B-cell lymphomas in 2 captive green tree pythons. CBCs in both cases revealed marked leukocytosis. Leukocytosis has been reported in cases of multicentric lymphoma in a diamond python (*Morelia spilota spilota*)^13^ and in red-tailed boas (*Boa constrictor constrictor*).^11,16^ For case 1, a presumptive diagnosis of lymphoma with leukemia was made when neoplastic round cells were identified within the lumen of several vessels. However, a definitive diagnosis of lymphocytic leukemia would require the identification of malignant cells in both the peripheral blood and bone marrow aspirates, which were not available for this case. Similar to mammals, lymphocytes in snakes are produced in the lymphoreticular system, and primary lymphopoietic sites include the fetal yolk sac, liver, and bone marrow.^8^ Reptilian lymphocytes are classified similarly to mammalian lymphocytes in terms of origin and function, and the overall count in peripheral blood is affected by temperature fluctuations, sex influences, and stress. Lymphocytosis can occur secondary to an inflammatory response, such as during parasitic, bacterial, and viral infections.^5^ The etiology of lymphoma is unknown in reptiles but may involve genetic and environmental factors, as well as infections.^3^

Lymphoma has been reported in various boids with concurrent reptarenaviral infection (boid inclusion body disease, BIBD; *Arenaviridae*, *Mammarenavirus*),^16,18^ and a round-cell tumor of unknown lineage was associated with ileocolic intussusception and enteritis in the same species.^11^ BIBD has been associated with various comorbidities, including neoplasia; however, the role of reptarenaviruses in initiating or promoting neoplasia remains unknown. We did not observe inclusions in H&E-stained tissue samples and blood smears in our 2 cases, and there has been no report of BIBD in the reptile collection at this zoologic institution. Molecular testing for BIBD was not performed for our 2 cases, as it is not currently available in Canada.

The main site of lymphoid proliferation in case 1 had some accompanying foci of B-cell inflammation. Luna stains were performed in an attempt to differentiate heterophilic from eosinophilic infiltrates. The granulocytes in the tumor contained brightly staining Luna-positive granules, consistent with eosinophils; however, Luna stains, widely used to identify eosinophils in mammals, have significant limitations in reptiles due to inconsistent staining patterns. Both heterophils and eosinophils can stain positively with the Luna method, reducing its reliability for distinguishing these granulocyte types in many reptilian species. Research in reptiles has shown that heterophils often have stronger staining intensity than eosinophils, which may not stain consistently. This overlap complicates the use of Luna staining as a definitive diagnostic tool in reptilian histology. Nonetheless, eosinophilic infiltrates are a common observation in lymphoma of other species due to production of eosinophil chemoattractants, such as IL5, by neoplastic lymphocytes. The eosinophils in the tumor in case 1 may reflect a similar process, and further support a diagnosis of T-cell lymphoma. Both cases underscore the importance of including neoplasms, such as lymphoma, as a differential diagnosis in cases of suspected granulomatous inflammation and, more broadly, in snakes with nonspecific clinical signs.

Lymphoma in reptiles remains a poorly understood disease with implications for both prognosis and management in captive settings. Although distribution was localized in case 1, it was widely disseminated in case 2, reflecting the variability in clinical progression. Disseminated lymphoma, as seen in case 2, likely carries a grave prognosis due to multi-organ involvement and systemic compromise. In contrast, localized lymphoma, as observed in case 1, might be amenable to surgical excision if detected early, potentially improving survival outcomes.

Treatment options for lymphoma in reptiles are largely extrapolated from mammalian oncology and include surgical intervention, chemotherapy, and supportive care.^3^ However, the lack of species-specific protocols poses significant challenges. Our 2 cases emphasize the need for early detection and thorough diagnostic workup, including histopathology and IHC, to guide decision-making.

Although immunohistochemical markers have become valuable tools in reptilian pathology, due to the lack of species-specific reagents, immunohistochemical characterization of neoplastic cells might be difficult, and results should be interpreted with caution. Most markers routinely used in veterinary diagnostic laboratories are validated and optimized for use in mammals. Although antibodies against the CD3 epsilon unit are reported to be reactive in most reptiles species to identify T cells,^5^ common markers for mammalian B cells (e.g., CD79a, CD20) often lack cross-reactivity.^1,8,15,20^ The PAX5 antigen, a nuclear transcription factor for B cells, is considered a reliable indicator of B-cell lineage in dogs, cats, and psittacine birds,^6^ and had strong nuclear immunolabeling in 2 cases of lymphoma in captive central bearded dragons (*Pogona vitticeps*).^15^ Our findings further corroborate that PAX5 is a good candidate marker for immunohistochemical characterization of lymphoma or leukemia in reptiles. Most of the neoplastic cells in case 2 had moderate to occasionally strong nuclear immunolabeling for PAX5, consistent with a B-cell lineage lymphoid neoplasm. Nuclear immunolabeling for PAX5 was also described in scattered neoplastic cells in case 1, often associated with granulocytic infiltration, supporting the use of this marker to detect cells of B lineage in CD3+ lymphomas. Additionally, IBA1, a monocyte/macrophage marker, was used in case 1 to exclude a histiocytic origin of the neoplastic cells. Strong cytoplasmic immunolabeling in numerous but isolated cells, interpreted as tumor-associated macrophages, was observed. Nonetheless, we found no reports of reliable use of this marker in reptiles, and results should be interpreted with caution.

We retrieved no cases of B-cell lymphoma in snakes, including green tree pythons (*Morelia viridis*), in a search of Google, PubMed, CAB Direct, Web of Science, and Scopus, using the search terms “B-cell lymphoma AND green tree python”, “B-cell lymphoma in green tree pythons”, “lymphoma AND green tree python”, “lymphoma AND Morelia viridis”, and “lymphoma AND snake”, suggesting that this condition has not been reported in this species. Although CD3+ T-cell lymphomas seem to be the most common type in snakes, neoplasms of B-cell origin have been diagnosed in Egyptian spiny-tailed lizards (*Uromastyx aegyptius*)^7^ based on light and electron microscopic features of the neoplastic cells and in a diamondback terrapin (*Malaclemys terrapin*) with acute lymphoblastic leukemia.^17^ In the latter report, the BLA36 antigen, a protein associated with malignant B cells, was detected using IHC in the cytoplasm of neoplastic cells.^17^ However, the same marker failed to label B cells in both normal resident populations in sections of gastrointestinal tract and spleen and in neoplastic cells in Egyptian spiny-tailed lizards diagnosed with lymphoid neoplasia.^7^ Despite the reported reliability of BLA36 to identify B cells in reptiles,^9^ the significance of IHC results needs to be interpreted conservatively in reptiles, as the reagents used for the procedure in both cited reports and our study were standardized for mammalian cell lines, and the normal lymphocyte lineages of reptiles remain poorly understood.
